# Treatment of limited stage follicular lymphoma with Rituximab immunotherapy and involved field radiotherapy in a prospective multicenter Phase II trial-MIR trial

**DOI:** 10.1186/1471-2407-11-87

**Published:** 2011-02-26

**Authors:** Mathias Witzens-Harig, Manfred Hensel, Michael Unterhalt, Klaus Herfarth

**Affiliations:** 1 Department of Internal Medicine V, University of Heidelberg, INF 410 69120 Heidelberg, Germany; 2 Onkologische Schwerpunkt Praxis, Q5 14-22, 68161 Mannheim, Germany; 3 Department Internal Medicine III, University of Munich (LMU), Marchioninistr. 15, 81377 Munich, Germany; 4 Department of Radiation Oncology, University of Heidelberg, INF 400; 69120 Heidelberg, Germany

## Abstract

**Background:**

The optimal treatment of early stage follicular Lymphoma is a matter of debate. Radiation therapy has frequently been applied with a curative approach beside watchful waiting. Involved field, extended field and total nodal radiation techniques are used in various protocols, but the optimal radiation field still has to be defined. Follicular lymphoma is characterized by stable expression of the CD20 antigen on the tumour cells surface. The anti CD20 antibody Rituximab (Mabthera^®^) has shown to be effective in systemic therapy of FL in primary treatment, relapse and maintenance therapy.

**Methods/design:**

The MIR (Mabthera^® ^and Involved field Radiation) study is a prospective multicenter trial combining systemic treatment with the anti CD20 antibody Rituximab (Mabthera^®^) in combination with involved field radiotherapy (30 - 40 Gy). This trial aims at testing the combination's efficacy and safety with an accrual of 85 patients.

Primary endpoint of the study is progression free survival. Secondary endpoints are response rate to Rituximab, complete remission rate at week 18, relapse rate, relapse pattern, relapse free survival, overall survival, toxicity and quality of life.

**Discussion:**

The trial evaluates the efficacy of Rituximab to prevent out-filed recurrences in early stage nodal follicular lymphoma and the safety of the combination of Rituximab and involved field radiotherapy. It also might show additional risk factors for a later recurrence (e.g. remission state after Rituximab only).

**Trial Registration:**

ClinicalTrials (NCT): NCT00509184

## Background

Worldwide, follicular lymphoma (FL) is the second most common type of non-Hodgkin's lymphoma (NHL) [1] with a rapidly increasing incidence in the last decades [2].. In the group of indolent lymphomas, FL is the most common type with 70% of cases. FL tumor cells are characterized by the translocation t (14;18)(q32;q21)and invariably express the CD20 antigen. The WHO Classification differentiates between grade I, II and III follicular lymphoma in respect to the number of centroblasts in the visual microscopy field. FL grade III is often regarded as an aggressive lymphoma and treated accordingly. Most patients are diagnosed in advanced disease stage and cannot be cured with conventional therapy. However, 15-30% patients are diagnosed in the limited Ann Arbor stages I and II and are potential candidates for a curative treatment approach. Standard of treatment for patients with limited disease with a curative intention is radiotherapy. However, no consensus of the required radiation field has been reached. Table 1 depicts the results of various radiotherapy trials in FL lymphoma in stage I and II. Most of the trials are retrospective analysis. Involved field (IF), extended field (EF) and total nodal (TN) irradiation techniques have been applied. The relapse free survival rate after 10 years was between 38% and 72% and the 10 year overall survival ranged in between 50% and 78%. Median survival was above 12 years, however, in some of the international trials which categorized lymphomas according to the Working Formulation also FL grade III and mantle cell lymphoma were part of the analysis. Forty-one percent of the patients had a relapse after 8 years in a prospective German observation trial with 117 patients with early stage FL who were treated with EF or TN. In stage I patients, there was no relapse in the irradiation field after 7 years, but 15% out of filed relapses. This trial led to the conclusion that large volume radiation techniques may prevent relapses. However, as extensive radiation protocols are associated with significant toxicities, grade 3 and grade 4 adverse events concerning the hematopoietic system were observed in 22% of patients [3].

There have been several studies which combined radiation therapy with systemic chemotherapy in early stage FL. Most studies failed to demonstrate a benefit of combined therapy [4-7]. In one study, the sequential administration of COP, CHOP-B and involved field irradiation improves relapse free survival, but not overall survival in comparison to historical cohort. Relapse free survival after 10 years was 72%, however 22% of patients experienced a grade IV neutropenia and 14 secondary malignancies were observed [8,9].

The monoclonal chimeric anti CD 20 antibody Rituximab has revolutionized the treatment of FL in the last decade. The pivotal phase II trial tested Rituximab monotherapy in 37 patients with refractory or relapsed FL. The overall response rate (ORR) was 46% with 8% complete responses (CR). The median time to progression (TTP) reached 10.2 months [10]. These promising results as well as other phase II clinical trials demonstrated a significant single agent activity of Rituximab in pretreated as well as in previously untreated patients with FL [11-13].. In combination with CHOP chemotherapy, Rituximab induced responses in all evaluable patients with a complete remission (CR) rate of 63% and a median PFS of 82 months in a Phase II trial [14]. These results were confirmed in four prospective randomised phase III studies investigating Rituximab in combination chemotherapy versus chemotherapy alone in first line therapy which showed significant increase in initial response rates, a significant prolongation of response duration and a significantly longer overall survival [15-18]. Rituximab was added to FCM (Fludarabine, Cyclophosphamde, Mitoxantrone) chemotherapy in relapsed FL in a prospective randomized Phase III trial and showed a significantly longer PFS and OS compared with FCM alone [19]. In addition to the impressing results of Rituximab as part of first line and relapse treatment, Rituximab maintenance therapy is effective in relapsed FL [20-22]. Rituximab maintenance therapy has also been shown to prolong PFS after first line therapy in two prospective randomized trials [23,24].

In the context of radiotherapy, the high efficacy of Rituximab in systemic treatment suggests that this drug may be of benefit in the eradication of minimal disease outside the radiation field. This concept is in line with the observation that Rituximab contributes to the elimination of minimal residual disease after cytotoxic therapy [25-27]. In addition, Rituximab may enhance radiosensitivity of lymphoma cells and may thus improve the efficacy of radiotherapy [28,29].

## Methods/Design

### Trial organization

The MIR trial has been designed by the Trial Center of the Department of Radiation Oncology and the Department of Internal Medicine in cooperation with the German Study Group for Low Grade Lymphoma (GLSG). The trial is an investigator initiated trial. Sponsor of the trial is the University Hospital of Heidelberg.

The trial is coordinated by the Department of Radiation Oncology of the University of Heidelberg in cooperation with the GLSG. The Dept. of Radiation Oncology is responsible for overall trial management, trial registration ClinicalTrials.gov Identifier: NCT00509184), database management, quality assurance including monitoring, reporting and for the scientific program of all trial related meetings.

A total of 15 centers in Germany participate in this trial. The centers are (listed alphabetically): Universityhospital Charité, Berlin; HELIOS Hospital, Berlin-Buch; Universityhospital Dresden, Universityhospital Essen; Universityhospital Göttingen; Universityhospital Hannover; Universityhospital Heidelberg; Universityhospital Cologne; Universityhospital Mainz; Universityhospital Mannheim; Universityhospital Marburg; Universityhospital Munich LMU; Universityhospital Munich TU; Universityhospital Ulm.

### On-site monitoring

During recruitment of patients monitoring on site is performed according to good clinical practice (GCP) guidelines. The data management will be performed by the Coordination Centre for Clinical Trials (KKS) of the University of Heidelberg.

### Ethics, informed consent and safety

The final protocol was approved by the ethics committee of the University of Heidelberg, Medical School (AFmu-085/2007, http://www.klinikum.uni-heidelberg.de) and the Paul-Ehrlich-Institute (PEI-registration number 432/06). This study complies with the Helsinki Declaration in its recent German version, the Medical Association's professional code of conduct, the principles of Good Clinical Practice (GCP) guidelines and the Federal Data Protection Act. The trial will also be carried out in keeping with local legal and regulatory requirements. The medical secrecy and the Federal Data Protection Act will be followed.

Written informed consent is obtained from each patient in oral and written form before inclusion in the trial and the nature, scope, and possible consequences of the trial have been explained by a physician. The investigator will not undertake any measures specifically required only for the clinical trial until valid consent has been obtained.

### Study design and endpoints

The MIR study is a prospective phase II study combining systemic Rituximab therapy with involved field irradiation in patients with FL WHO Grad 1 and 2 in Stage I and II.

This trial aims at testing the combination's efficacy and toxicity with an accrual of 85 patients.

Primary endpoint of the study is progression free survival (2 years). Secondary endpoints are complete remission rate after Rituximab monotherapy (week 7) and after complete treatment (week 18), relapse rate, relapse pattern, relapse free survival, overall survival, toxicity and quality of life.

### Patient selection

In order to be included in the MIR trial, patients (18 to 75 years) are required to have pathologically documented FL Who grade 1 or 2 in Ann Arbor Stage I or II. The histology has to be confirmed by one of the reference pathologists of the GLSG. Evaluation of CD20 status is compulsory. Maximal tumour diameter is 7 cm. Accrued patients will be expected to demonstrate sufficient compliance, they should also live in relative proximity of the centre of care to ensure adequate follow-up after treatment. In addition, adequate haematological function is essential for inclusion in the trial (leukocytes ≥ 3000 × 10^3 ^/ml, platelets ≥ 100000 × 10 ^3 ^/ml, and haemoglobin ≥ 10 g/dL). Patients are required to use adequate contraception during and at least up to 3 months after treatment. Naturally, written informed consent is obtained prior to commencement of treatment in this trial.

Patients are not eligible with previous irradiation, chemotherapy of immunotherapy. Patients with severe concurrent systemic disease or other malignant disease (apart from cervical carcinoma in-situ, basal cell carcinoma, or unless previously treated and in remission for ≥ 5 years without further treatment) can also not be included in this trial. Furthermore, clinical performance worse than ECOG 2, bulky disease (> 7 cm), splenic involvement, immunodeficiency, viral hepatitis, collagenosis, severe psychiatric illness, hypersensitivity to foreign proteins will prohibit inclusion as will pregnancy or breast feeding.

### Statistical Design

The hypothesis states an improvement of the progression free survival after 2 years from 75% to 89% (risk reduction by 40% using Rituximab). 77 patients have to be evaluated to proof the hypthesis with a 95% power using a single arm binomial test. An additional 8 patients have to be recruited for a possible 10% drop-out rate.

### Work up

Patients with pathologically documented nodal FL Who grade 1 or 2 in stage I or II receive a complete work-up including, cervical, thoracic, abdominal and pelvic CT scans, bone marrow histology and extensive laboratory testing. An ENT examination is mandatory for supra diaphragmal disease and a kidney function test for abdominal disease. The FLIPI risk score will be calculated for each patient. All in- and exclusion criteria are examined. In the event of patients meeting the required inclusion criteria, information about participation in the study with possible risks and benefits is given to the patients and written informed consent is obtained. In the event of patients declining treatment within the MIR trial, irradiation alone is offered.

### Treatment

**Figure 1:**
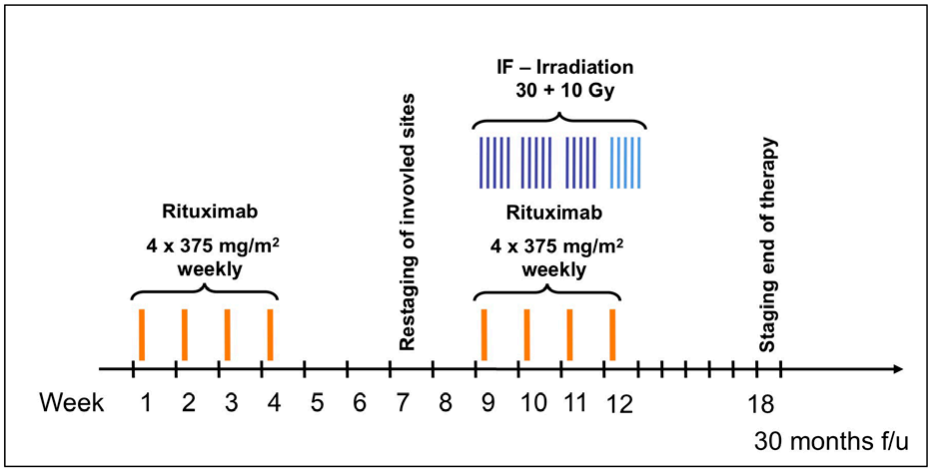
**Study Schema of the MIR trial (IF = involved-field; f/u = follow up)**.

The treatment lasts over 12 weeks and is divided in 3 sections as illustrated in Figure 1. The first section consists of 4 weekly i.v. infusions of Rituximab (Roche AG, Basel, Switzerland) at a standard dosage of 375 mg/m^2 ^given on day 1, 8, 15 and 22. There is a 4 week treatment break thereafter (week 5 - 8) which includes a radiation therapy planning CT of the involved body region in week 7. Response to the Rituximab therapy is also evaluated at this time. Another 4 weekly infusions of Rituximab are administered in week 9-12. At the same time, radiation therapy of the involved lymph node region is applied, a three dimensional radiation planning is compulsory for all patients having abdominal or retroperitoneal disease. Involved field irradiation consists of a dose of 30 Gy in daily fractions of 2 Gy (Monday to Friday). Patients who still show enlarged lymph nodes in week 7 receive additional boost irradiation with a total dose of 40 Gy. Accepted tolerance doses of organs at risk must not be exceeded.

### Safety and discontinuation of treatment

Toxicities are classified by grade, type, duration, onset, and relationship to study treatment.

Treatment of Rituximab-induced adverse reactions is carried according to recommendations by the provider. All patients will receive anti allergic prophylaxis (paracetamol, clemastin) and when appropriate, corticosteroids. In the case of fever (> 38,5°C), chills, bronchial constriction, hypotension (> 30 mm Hg) it is recommended to discontinue treatment with Rituximab.

Analysis of safety related data is performed with respect to frequency of:

• Serious Adverse Events and Adverse Events stratified by organ-system

• Adverse Events stratified by severity

• Adverse Events stratified by causality.

Patient toxicities will be assessed using the NCI Common Toxicity Criteria (CTC). Toxicity will be evaluated prior to treatment, weekly prior to each course of infusional Rituximab and at follow-up. Unacceptable toxicity is defined as unpredictable, or irreversible Grade 4 toxicity. Decisions regarding Rituximab dose-adjustment will be made using the guidelines below and based on haematological parameters (ANC and platelets) monitored weekly during radiation before each dose of Rituximab.

Whenever necessary, metoclopramide or 5-HT_3_-antagonists are used for antiemesis.

Antihistamines such as clemastine are administered prior to Rituximab application.

### Trial duration

Patient recruitment is planned to be completed after 24 months. Patients will be monitored for two and a half years after study entry. The total duration of the trial is estimated to be 4 and a half years. Recruitment started in March 2008. As of February 2010, 65 patients have been included in the trial.

Individual exclusion criteria are serious adverse reactions or the patient's voluntary withdrawal from the trial.

### Assessment of therapeutic efficiency

CT or MRI of the involved regions are scheduled for week 7 and 18. For screening of the whole body stem, CT or MRI imaging is planned for months 6, 12, 18, 24 and 30 after start of therapy. At all above mentioned follow-up visits, there is also a clinical examination with palpation of all lymph node sites schedulded.

Local response is evaluated in accordance with the Cheson Criteria [30].

• **Complete response (CR) **is the complete disappearance of all detectable evidence of disease on CT, and all disease-related symptoms, and normalization of biochemical abnormalities, and normal bone marrow biopsy (BMB). Previously involved nodes on CT more than 1.5 cm in their greatest axial diameter must regress to less than 1.5 cm, and previously measured nodes of 1.1-1.5 cm must decrease to less than 1 cm.

• **CRu (uncertain) **corresponds to CR criteria but with a residual mass more than 1.5 cm in greatest axial diameter that has regressed by more than 75%.

• **Partial response (PR) **is at least 50% reduction in the sum of the product of the greatest diameters (SPD) of the six largest nodes with no increase in the size of other nodes and no new sites of disease. Splenic and hepatic nodules must regress by at least 50% in the SPD.

• **Stable disease (SD) **is less than a PR but is not progressive disease. Progressive disease (PD) is more than 50% increase in the sum of the product of the greatest diameters of any previously abnormal node, or appearance of any new lesions during or at the end of therapy.

• **Relapsed disease (RD) **is the appearance of any new lesion or increase in size of more than 50% of previously involved sites or nodes in patients who achieved CR or CRu.

### Quality assurance program

All staging examinations are reviewed centrally as part of the quality assurance program. Completeness of the staging, quality of the imaging and extend of involvement are analysed. In addition, a proposal for the radiation site is sent out to the treatment centre. All radiation treatment plans are also centrally reviewed in a retrospective manner.

## Discussion

The MIR trial is evaluating the effectiveness and the safety of the combination of an involved field radiation therapy with the systemic anti CD20 antibody Rituximab for early stage follicular lymphoma grade 1 or 2 in a multi center setting.. It also might show additional risk factors for a later recurrence (e.g. remission state after Rituximab only).

## Competing interests

The trial is funded by Roche Pharma AG, Switzerland. Funding includes trial organization and monitoring by the KKS Heidelberg, the statistical analysis, data management and the supply of the study medication. There is no other funding of the trial.

The authors have no financial relationship with Roche Pharma AG. The authors also participate in other scientific trials which are supported by Roche Pharma AG.

## Authors' contributions

KKH and MH planned the study. KKH and MWH coordinated and conducted the study and wrote the manuscript. MU was responsible for the statistical design.

## Pre-publication history

The pre-publication history for this paper can be accessed here:

http://www.biomedcentral.com/1471-2407/11/87/prepub
